# γ‑Ray, Charged Particle, and Neutron Attenuation Characteristics of Cadmium-Containing 211 MAX Phase Carbides, M_2_CdC (M = Ti, Zr, and Hf)

**DOI:** 10.1021/acsomega.5c04825

**Published:** 2025-10-21

**Authors:** Celal Avcıoğlu

**Affiliations:** Faculty of Fine Arts, Ceramic Department, Usak University, 64000 Usak, Turkey

## Abstract

This study examines the attenuation behavior of γ rays and both charged (proton, α, electron) and uncharged (neutron) particles in cadmium-containing Group 4 MAX phase carbides: Ti_2_CdC, Zr_2_CdC, and Hf_2_CdC. Key parameters such as the linear attenuation coefficient (LAC), atomic cross section (ACS), electronic cross section (ECS), half-value layer (HVL), mean free path (MFP), effective atomic number (*Z*_eff_), and energy absorption buildup factor (EABF) were evaluated for γ-ray interactions. Among the studied materials, Hf_2_CdC, with a density of 12.67 g/cm^3^, exhibited superior γ-ray shielding performance, reflected in its high LAC and low HVL and MFP. Its effectiveness is comparable to that of conventional shielding materials such as lead (Pb). Notably, Hf_2_CdC demonstrated an HVL of 0.968 cm at 1173 keV, outperforming Pb (0.990 cm) at the same energy. This enhanced performance at γ energies dominated by Compton scattering is primarily attributed to its high density. For charged particles, Ti_2_CdC showed the highest mass stopping power for protons and α particles. However, due to its higher density, Hf_2_CdC resulted in the shortest penetration depths; for example, 10 MeV protons had a penetration range of only 1.77 μm in Hf_2_CdC. Additionally, Hf_2_CdC exhibited a high fast neutron removal cross section (FNRCS) of 0.163 cm^–1^, significantly outperforming common neutron shielding materials such as B_4_C, graphite, and concrete. Furthermore, all three compounds demonstrated excellent thermal neutron absorption due to the presence of cadmium (Cd), with Hf_2_CdC achieving the highest thermal neutron LAC of 43.38 cm^–1^. These results identify Hf_2_CdC as a strong candidate for integrated, multipurpose radiation shielding applications.

## Graphical abstract

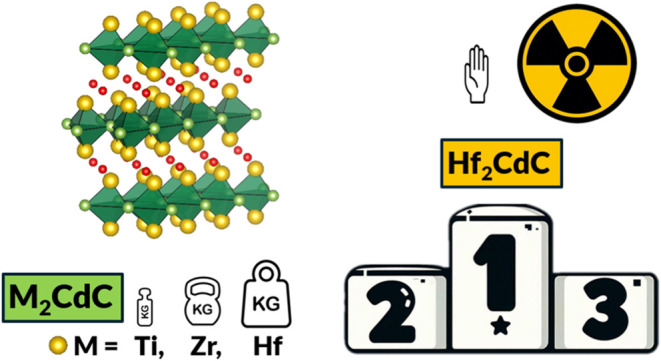

## 1 Introduction

Radiation shielding is essential for protecting living organisms, sensitive equipment, and the environment from the harmful effects of ionizing radiation.^1,2^ The expanding use of ionizing radiation in nuclear energy, materials science, medical imaging, and space exploration has significantly increased the demand for advanced shielding materials. Depending on the application, these materials should effectively attenuate one or more types of radiation, such as γ rays, charged particles, and neutrons. In addition to attenuation performance, practical shielding applications require materials with robust engineering properties, such as mechanical strength, thermal stability, and long-term durability, especially under harsh radiological conditions.^3–5^

γ Rays are high-energy electromagnetic radiation, similar to X-rays but emitted from the atomic nucleus during radioactive decay or nuclear reactions.^6^ Conventional γ-ray shielding materials typically contain high atomic number elements such as Pb, tungsten, or bismuth.^7–12^ Their effectiveness results from high electron densities and strong nuclear charges, which increase the likelihood of γ-ray interactions through the photoelectric effect, Compton scattering, and pair production.^13^

Conversely, neutron radiation, composed of uncharged subatomic particles demands vastly different material properties for efficient attenuation. Neutron shielding generally involves two stages. First, fast neutrons are moderated to thermal energies through collisions with atomic nuclei. This moderation is effectively achieved using materials rich in hydrogen (e.g., polymers or water), boron (e.g., B_4_C), or carbon (e.g., graphite).^14–16^ These materials are effective because their light nuclei maximize energy transfer during elastic scattering. This is particularly true for hydrogen, whose proton has nearly the same mass as a neutron, enabling effective reduction of neutron kinetic energy. Once thermalized, neutrons are captured by materials with high thermal neutron capture cross sections, such as boron, Cd, samarium, dysprosium, or gadolinium.^17–21^

In addition to elemental composition, material density plays a critical role in radiation attenuation. Higher density means a greater number of atoms or nuclei per unit volume, which directly increases the probability of interaction with incident radiation, whether photons or particles.^22,23^ The achievable density of a material is closely related to its crystal structure. Indeed, crystal structures that enable high atomic packing efficiency can yield materials with greater mass per unit volume. For instance, crystal structures with high packing factors accommodate more mass within a given volume than to more open structures composed of elements with similar atomic weights. Therefore, when developing novel shielding materials, it is essential to consider both the constituent elements and atomic packing efficiency to achieve effective radiation attenuation without excessive thickness.

MAX phases are a family of layered ternary compounds first discovered in the 1960s and have attracted renewed attention since the 1990s.^24^ They possess a general formula of M_*n*+1_AX_*n*_ (*n* = 1–4), where M is an early transition metal, A is an A-group element (commonly from Group 13, 14, and 15), and X is carbon, nitrogen, boron, or phosphorus. Depending on the value of *n*, MAX phases are categorized into groups such as 211 (*n* = 1), 312 (*n* = 2), and 413 (*n* = 3).^25^ These phases crystallize in either orthorhombic or hexagonal symmetry. The latter one, in particular, enables efficient atomic packing, which contributes to high material densities that are important for interactions with energetic radiation.

A distinctive feature of MAX phases is their unique combination of metallic and ceramic characteristics. They exhibit high-temperature strength, resistance to oxidation and corrosion, thermal shock resistance, and excellent tolerance to damage and radiation.^26–31^ These properties make them attractive for use in extreme environments. In addition, their wide compositional flexibility across the M, A, and X sites allows for precise tuning of physical, chemical and nuclear properties. With the number of known MAX phases now approaching 400,^25^ and with synthesis routes such as solid-state reactions, molten salt, physical vapor deposition, and hot pressing being actively developed for new compositions,^32,33^ this rapidly expanding family of materials is being actively explored for a broad range of advanced technological applications.

Despite these advantages, the radiation shielding potential of MAX phases remain largely unexplored. Within this family, M_2_CdC (*n* = 1) compounds, which crystallize in hexagonal symmetry and contain Cd at the A-site are particularly promising candidates for multifunctional radiation shielding materials. The composition of M_2_CdC MAX phases combines low atomic number carbon at the X-site with medium atomic number Cd (*Z* = 48) and M-site elements such as titanium (Ti, *Z* = 22), zirconium (Zr, *Z* = 40), and hafnium (Hf, *Z* = 72). This hybrid configuration supports broad-spectrum attenuation of γ rays, neutrons, and charged particles. High atomic number elements enhance γ-ray and particle interaction, while light elements like carbon and Ti contribute to neutron moderation. Cd further enhances the design by effectively capturing thermal neutrons as Cd is a well-known thermal neutron absorber due to its high neutron capture cross-section, approximately 2450 barns.^34^ Note that, while Cd provides excellent thermal neutron capture, its known toxicity and environmental impact require careful handling and potential encapsulation.^35^ Incorporating Cd into stable compounds such as MAX phases may help mitigate these risks while preserving its neutron-absorbing capability.

There is growing interest in materials that can attenuate multiple forms of radiation while maintaining structural performance, thermal stability, and long-term reliability. Conventional shielding materials often provide only one of these functions and therefore require multilayered systems to meet diverse radiation protection requirements.^36–38^ This study aims to address this limitation by evaluating the radiation attenuation capabilities of M_2_CdC compounds with different M-site elements, Ti, Zr, and Hf. The objective is to demonstrate the potential of these compounds as multifunctional shielding materials and to provide insights into how variations in atomic number and material density influence shielding performance against γ rays, charged particles (protons, α particles, electrons), and neutrons. These findings may help guide the design of next-generation shielding solutions based on MAX phase materials.

## 2 Methods

The radiation attenuation behavior of M_2_CdC (M = Ti, Zr, and Hf) MAX phase carbides was investigated using computational approach. Structural properties and densities for Ti_2_CdC were obtained from experimental measurements,^39^ whereas those for Zr_2_CdC and Hf_2_CdC were taken from previous Density Functional Theory (DFT) study.^40^ These parameters, summarized in Table 1, served as inputs for all calculations.

**Table 1:** Structural and Calculated Physical Parameters of M_2_CdC (M = Ti, Zr, and Hf) Max-Phases

compound	Ti_2_CdC	Zr_2_CdC	Hf_2_CdC
M-element (Z)	22	40	72
weight fractions of M-site elements (%)	43.48	59.45	74.15
space group	*P*6_3_/*mmc* (No. 194)	*P*6_3_/*mmc* (No. 194)	*P*6_3_/*mmc* (No. 194)
lattice parameter *a (*Å)	3.099	3.226	3.176
lattice parameter *c (*Å)	14.41	14.839	14.453
*c*/*a*	4.65	4.59	4.55
unit cell volume *V* (Å^3^)	119.8	133.7	126.2
molar mass (g/mol)	220.15	306.87	481.4
calculated density (g/cm^3^)	6.10	7.63	12.67

The γ-ray shielding parameters, including the mass attenuation coefficient (MAC), linear attenuation coefficient (LAC), half-value layer (HVL), mean free path (MFP), effective atomic number (*Z*_eff_), atomic cross section (ACS), electronic cross section (ECS), and energy absorption buildup factor (EABF) were all computed using the Phy-X/PSD platform.^41^ This software utilizes photon interaction cross sections from the XCOM database^42^ and enables calculations across the photon energy range of 15 keV to 15 MeV. Unless otherwise noted, a narrow-beam geometry was assumed for attenuation parameters (MAC, LAC, HVL, and MFP), while EABF was obtained using the Geometric Progression (G-P) fitting method, which inherently accounts for scattering in broad-beam conditions. The computational approach has been previously validated by comparing Phy-X/PSD–calculated γ-ray attenuation coefficients with experimental measurements in our earlier study, showing negligible differences and confirming the reliability of the present simulations.^13,22^

The mass attenuation coefficient (MAC, μ/ρ) quantifies the probability of photon interaction per unit mass of a material and is fundamentally based on the Beer–Lambert law

1 $$I=I_{0}e^{-\mu x}$$

where *I*_0_ and *I* are the incident and transmitted photon intensities, respectively, μ is the LAC, and *x* is the material thickness. The MAC can be expressed as

2 $$\mu_{m}=\frac{1}{\rho t}\mathrm{In}\left(\frac{l_{0}}{l}\right)$$

with ρ representing the material density. For composite materials, the MAC is calculated using the elemental mixture rule

3 $$\mu_{m}=\sum_{i}w_{i}{\left(\frac{\mu }{\rho }\right)}_{i}$$

where, *w*_*i*_ is the weight fraction of the *i*th element.

The linear attenuation coefficient (LAC, μ) is related to the MAC by the density of the material

4 $$\mu =\mu_{m}\times \rho$$

HVL can be defined as the material thickness required to reduce the photon intensity to one-half of its original value. The HVL can be expressed as

5 $$\mathrm{HVL}=\frac{\mathrm{In}\left(2\right)}{\mu }=\frac{0.693}{\mu }$$

Similarly, the MFP, representing the average distance a photon travels before an interaction occurs, can be expressed as

6 $$\mathrm{MFP}=\frac{1}{\mu }$$

The *Z*_eff_ of a compound or mixture can determined from the ACS and ECS.

7 $$Z_{\mathrm{eff}}=\frac{\sigma_{a}}{\sigma_{e}}$$

The ACS (σ_a_) can be obtained from the MAC as

8 $$\sigma_{a}=\frac{N_{A}}{A}\mu_{m}$$

where *N*_A_ is the Avogadro constant.

The ECS (σ_e_) can be calculated using the relation

9 $$\sigma_{e}=\frac{1}{N_{A}}\left(\sum_{k}\frac{f_{i}A_{i}}{Z_{i}}{\left(\mu_{m}\right)}_{i}\right)=\frac{\sigma_{a}}{Z_{\mathrm{eff}}}$$

where *Z*_*i*_, *f*_*i*_ and *A*_*i*_ is the atomic number, mole fraction and atomic weight of the *i*th element.

Additionally, EABF was calculated using the well-established Geometric Progression (G-P) fitting method,^43^ with fitting coefficients (*a*, *b*, *c*, *d*, and X_k_) obtained from the ANSI/ANS-6.4.3 database.^44^ Further details on the theoretical basis and computational procedure can be found in the Phy-X/PSD reference.^41^

To assess the neutron attenuation capability, the FNRCS (∑*_R_*) was calculated using the Phy-X/PSD platform according to the relation

10 $$\sum_{R}={\sum_{i}\rho_{i}{(\Sigma }_{R}/\rho )}_{i}$$

where ρ_*i*_ is the partial density of the *i* ^th^ constituent, and (∑_*R*_/ρ)_i_ is the mass removal cross-section.

In addition, the thermal neutron linear attenuation coefficient was evaluated using the NGCal tool,^45^ which relies on isotopic cross-section data at a thermal neutron energy of 25.4 meV.

The fundamental parameters governing charged particle transport were simulated over an energy range from 0.01 to 10 MeV. For protons (H^+^) and α particles (He^2+^), the electronic, nuclear, and total mass stopping powers, along with the penetration depth (range), were calculated using the Stopping and Range of Ions in Matter (SRIM) code.^46^ For electrons, the collision, radiative, and total mass stopping powers, as well as the penetration depth, were determined using the Electron Stopping Powers and Ranges (ESTAR) database.^47^

## 3 Results and Discussion

Figure 1 presents the LAC of the three Cd-containing MAX phases (Ti_2_CdC, Zr_2_CdC, and Hf_2_CdC) as a function of incident photon energy. The LAC, which quantifies a material’s ability to attenuate γ rays, shows a strong dependence on photon energy for all three compositions. At lower photon energies (below approximately 0.1 MeV), the LAC values are substantially higher, indicating a greater probability of photon interaction and enhanced attenuation in this energy range. As the incident photon energy increases, the LAC decreases rapidly, indicating that higher-energy photons interact less and are more likely to penetrate the material. This trend reflects the energy-dependent nature of γ-ray interactions: the photoelectric effect dominates at low energies, while Compton scattering becomes more prominent at intermediate energies.

**Figure 1:**
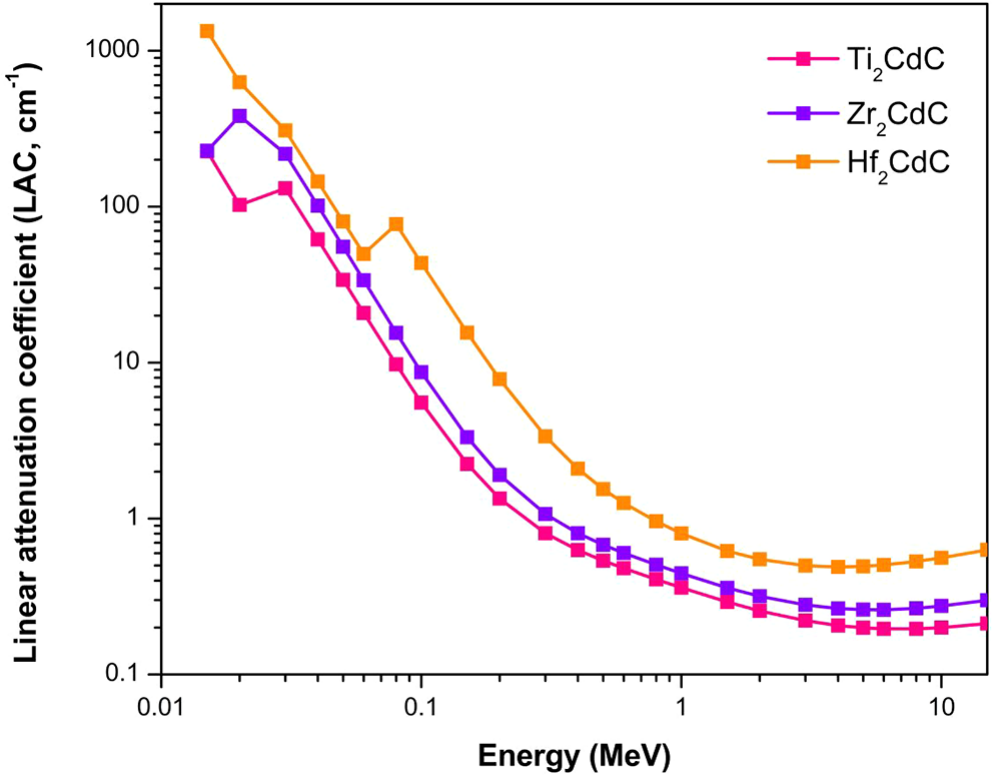
Variation of linear attenuation coefficient with incident photon energy for the Ti_2_CdC, Zr_2_CdC, and Hf_2_CdC MAX phases.

Throughout the entire energy range investigated, Hf_2_CdC consistently demonstrates the highest LAC values, indicating the strongest γ-ray shielding performance among the studied MAX phases. Zr_2_CdC shows intermediate LAC values, while Ti_2_CdC has the lowest, suggesting the weakest attenuation.

The superior LAC of Hf_2_CdC is primarily attributed to the higher atomic number of Hf (*Z* = 72) compared to Zr (*Z* = 40) and Ti (*Z* = 22) especially in the low-energy region. The probability of photoelectric absorption, which is the dominant interaction mechanism at these energies, depends strongly on the atomic number. Furthermore, the higher density of Hf_2_CdC compared to Zr_2_CdC and Ti_2_CdC also contributes to a greater number of atoms per unit volume, thereby increasing the overall probability of photon interaction and resulting higher LAC.

To gain deeper insights into the fundamental interaction probabilities between γ photons and the constituent atoms and electrons of the MAX phases, ACS and ECS were examined as a function of incident photon energy (Figure 2). The ACS represents the effective cross-sectional area an atom presents for interaction with an incident photon and directly indicates the interaction probability per atom. In contrast, the ECS quantifies the average contribution of each electron to the atom’s total photon interaction probability. Both parameters show a general decrease in interaction probability as photon energy increases, consistent with the LAC results. Notably, the ACS starts at approximately 10^–18^ cm^2^ per atom at 0.015 MeV, and decreases by about 5 orders of magnitude, reaching the 10^–23^ cm^2^ per atom range at 10 MeV. Superimposed on this overall decline are abrupt rises, especially at lower energies, which correspond to the specific absorption edge energies of the constituent atoms, as listed in Table 2.

**Figure 2:**
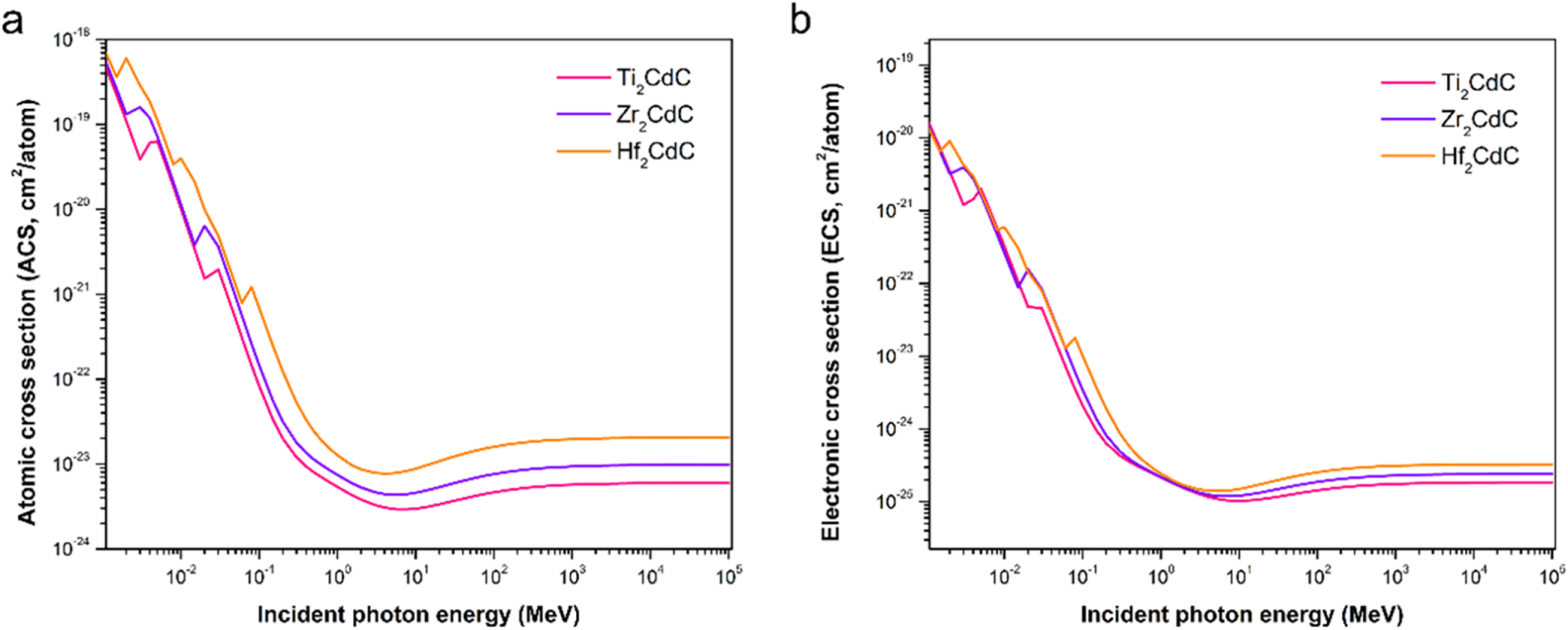
Variation of (a) atomic cross section and (b) electronic cross section with incident photon energy for the Ti_2_CdC, Zr_2_CdC, and Hf_2_CdC MAX phases, illustrating the per-atom and per-electron interaction probabilities with γ-ray photons.

**Table 2:** X-ray Absorption Edge Energies of C, Cd, Ti, Zr and Hf Elements in KeV^a^

elements	K-edge	L1-edge	L2-edge	L3-edge	M1-edge	M5-edge
C	0.283	—	0.006	0.006	—	—
Cd	26.711	4.018	3.727	3.537	0.772	0.405
Ti	4.966	0.563	0.461	0.455	0.058	—
Zr	17.997	2.531	2.306	2.222	0.430	0.178
Hf	65.351	11.271	10.739	9.561	2.601	1.662

a Adapted with permission from ref 48 Copyright 1967, American Physical Society.

These absorption edges correspond to the characteristic energies at which the photoelectric cross-section exhibits a pronounced increase, occurring when the incident photon energy surpasses the binding energy of core–shell electrons (e.g., K- and L-shells). At these threshold energies, photons possess sufficient energy to eject tightly bound electrons via the photoelectric effect, resulting in a discrete jump in the atomic absorption cross-section. Due to the higher atomic number of Hf, its K- and L-edge binding energies are situated at higher photon energies relative to Zr and Ti. This shift elevates the atomic cross-section of Hf-containing MAX phases across a broader energy range, thereby enhancing the overall macroscopic linear attenuation coefficient of Hf_2_CdC.

The ECS, similarly decreases with increasing photon energy, but remains two to three orders of magnitude lower than the ACS, typically ranging from approximately 10^–21^ cm^2^ per electron at 0.015 MeV to about 10^–25^ cm^2^ per electron at 10 MeV for Hf_2_CdC.

A notable trend evident in Figure 2 is the consistent ordering of both ACS and ECS values across the examined energy range: Hf_2_CdC > Zr_2_CdC > Ti_2_CdC. This sequence directly reflects the increasing atomic numbers of the M-site elements (Hf = 72, Zr = 40, Ti = 22), highlighting the significant impact of the M-site atomic properties on fundamental photon interaction probabilities. The higher atomic number corresponds to an increased number of electrons per atom and enhances the likelihood of photon interactions on both a per-atom and per-electron basis, especially in energy regions dominated by the photoelectric effect and pair production. However, the smaller separation in the ECS curves compared to the ACS curves suggests that, while total electron count affects overall interaction probability, the per-electron interaction probability is less sensitive to changes in the M-site element. In contrast, the per-atom interaction probability remains more closely linked to atomic number and electron configuration.

The energy dependent γ-ray shielding characteristics of the MAX phases are further clarified by examining their *Z*_eff_ and the ratio of incoherent to total scattering (with coherent), as shown in Figure 3. The *Z*_eff_ is a key parameter for compounds, representing their average atomic behavior toward γ rays as if they were a single element. It helps predict and compare the shielding effectiveness of materials.

**Figure 3:**
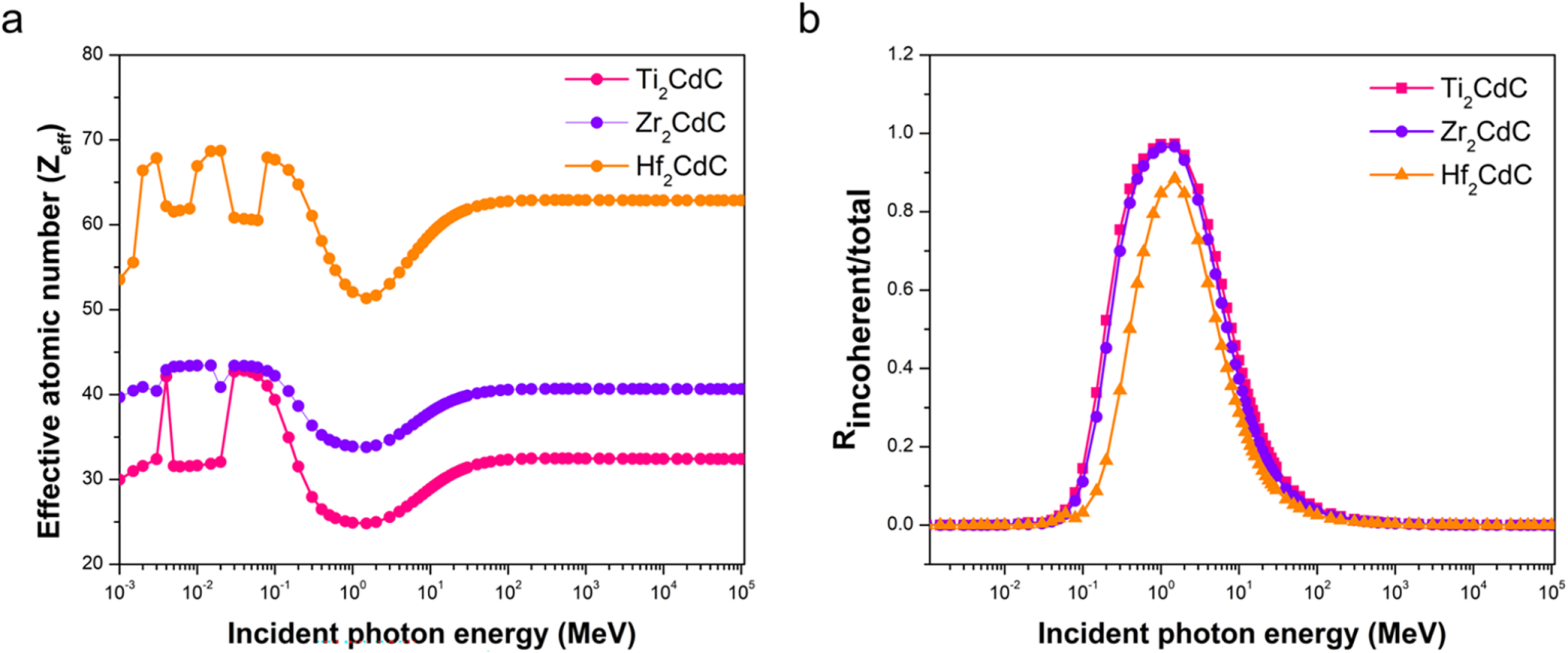
Variation of (a) effective atomic number (*Z*_eff_), and (b) the ratio of incoherent to total scattering (*R*_incoherent/total_) with the incident photon energy for the investigated Cd-containing 211-MAX phases.

As shown in Figure 3a, Hf_2_CdC consistently exhibits the highest *Z*_eff_ across the energy range, starting between 54 and 70 at lower energies (below 0.1 MeV) and then stabilizing around 63 at higher energies (above approximately 3 MeV). Zr_2_CdC displays intermediate *Z*_eff_ values, ranging roughly from 39 to 44 below 0.1 MeV before settling near 40 at higher energies. Ti_2_CdC has the lowest *Z*_eff_, varying from 30 to 42 at lower energies and stabilizing around 30 at higher energies. The fluctuations at lower energies arise from the X-ray absorption edges of the constituent elements (Table 2), which enhance photoelectric absorption and thus affect the effective atomic response. The lowest *Z*_eff_ values for each sample are generally occur in the intermediate energy region (approximately 0.1 to 4 MeV), where Compton scattering dominates, with approximate values of 25 for Ti_2_CdC, 33 for Zr_2_CdC, and 51 for Hf_2_CdC. The photon energies corresponding to these minimum *Z*_eff_ values also coincide with the highest HVL and MFP, indicating the materials’ poorest shielding effectiveness against photons in this energy range.

Figure 3b shows the ratio of incoherent to total scattering for the investigated MAX phases as a function of incident photon energy. This ratio highlights the relative contributions of Compton (incoherent) versus coherent scattering during γ-ray interactions. The ratio varies between 0 and 1, with values closer to 1 indicating a dominance of incoherent scattering. Across the examined energy spectrum, all three MAX phases exhibit a prominent peak in the ratio of incoherent to total scattering between approximately 0.1 and 10 MeV, with maxima occurring around 1 MeV. This indicates that Compton scattering is the dominant scattering mechanism in this in this range, significantly outweighing coherent scattering. The peak ratios reach approximately 0.95 for Ti_2_CdC, 0.98 for Zr_2_CdC, and 0.88 for Hf_2_CdC.

At lower energies (below approximately 0.01 MeV), the ratio of incoherent to total scattering approaches zero for all three materials. This behavior reflects the dominance of photoelectric absorption at these energies, which greatly reduces the likelihood of scattering events. Similarly, at higher energies (above approximately 10 MeV), the ratio also declines toward zero. This decrease results from the increasing significance of pair production (an absorption mechanism), which also lowers the overall probability of scattering.

Interestingly, at the 1 MeV energy level where Compton scattering dominance reaches its peak, the peak values show a subtle dependence on the constituent elements. Hf_2_CdC, containing the highest atomic number element (Hf), exhibits the lowest ratio of incoherent to total scattering, while Zr_2_CdC and Ti_2_CdC, with lower atomic number elements, show slightly higher values. This observation suggests that a higher atomic number may slightly reduce the relative probability of Compton scattering.

HVL and MFP are fundamental parameters in evaluating a material’s effectiveness in attenuating ionizing radiation. HVL represents the thickness of a material required to reduce the intensity of incident radiation by half, offering a direct measure of shielding efficiency. A lower HVL indicates better attenuation performance. Meanwhile, MFP denotes the average distance a photon travels within a material before interacting with it, reflecting the likelihood of photon–matter interactions. Figure 4 presents HVL and MFP for the three MAX phases as a function of incident photon energy.

**Figure 4:**
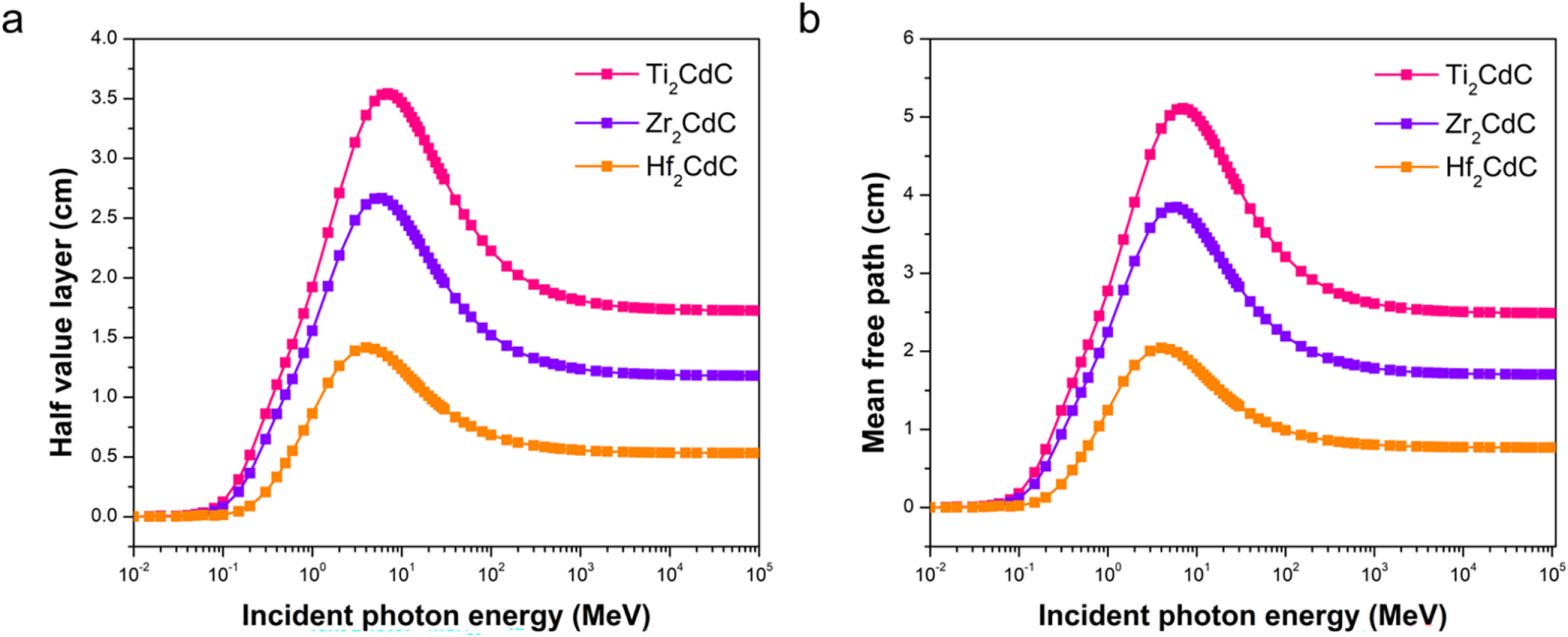
Variation of (a) half-value layer and (b) mean free path with incident photon energy for the Ti_2_CdC, Zr_2_CdC, and Hf_2_CdC MAX phases.

As observed in Figure 4a, the HVL for all three MAX phases initially increases with increasing photon energy, reaches a peak, and then decreases slightly. This indicates that the penetration power of γ rays increases initially, but at very high energies, other interaction mechanisms become more significant, leading to a slight decrease in HVL. Consistently, Hf_2_CdC exhibits the lowest HVL across the energy range, confirming its superior shielding ability. Ti_2_CdC has the highest HVL, indicating the poorest shielding performance, while Zr_2_CdC falls between the two.

The MFP follows a trend similar to that of the HVL, as shown in Figure 4b. This parallel behavior reflects their shared dependence on the underlying photon attenuation mechanisms discussed earlier. Notably, the trends observed in both HVL and MFP are consistent with the trends in LAC (Figure 1), where Hf_2_CdC, with higher LAC values has lower HVL and MFP values, indicating more effective attenuation of γ rays. The differences in HVL and MFP among the three MAX phases are attributed to their varying elemental compositions. Hf_2_CdC, with the highest density and atomic number element (Hf), exhibits the best shielding performance (lowest HVL and MFP) due to the higher probability of photon interactions.

To further contextualize the shielding performance of Hf_2_CdC, its HVL and MFP values are compared with those of several conventional and advanced shielding materials, as summarized in Table 3. Across the investigated photon energies (662, 1173, and 1332 keV), Hf_2_CdC consistently exhibits substantially lower HVL and MFP values than commonly used materials such as PbO-SiO_2_ glass (1:1 molar ratio), ordinary and heavy Barite concretes, and industrial alloys including Inconel 600, Inconel 625, and Stainless Steel 304. This difference highlights the superior attenuation efficiency of Hf_2_CdC per unit thickness, indicating that a smaller amount of material is required to achieve an equivalent level of radiation protection.

**Table 3:** Calculated HVL and MFP Values for the M_2_CdC (M= Ti, Zr, Hf) MAX Phase Carbides, in Comparison with Selected Conventional γ-Ray Shielding Materials, at Photon Energies of 662, 1173, and 1332 keV

		half value layer (cm)			mean free path (cm)		
sample	density (g/cm^3^)	662 keV	1173 keV	1332 keV	662 keV	1173 keV	1332 keV
Ti_2_CdC	6.10	1.536	2.106	2.252	2.216	3.038	3.248
Zr_2_CdC	7.63	1.22	1.70	1.82	1.77	2.45	2.62
Hf_2_CdC	12.67	0.61	0.97	1.05	0.88	1.39	1.51
PbO-SiO_2_ glass (1:1 mol)^49–51^	6.00	1.11	1.87	2.13	1.60	2.70	3.07
concrete^49,52–54^	2.30	3.85	5.09	5.36	5.55	7.34	7.73
barite concrete^49,52–54^	3.35	2.65	3.69	3.98	2.83	5.33	5.74
Inconel-600 ^49^	8.47	1.10	1.46	1.56	1.58	2.11	2.25
Inconel-625 ^49^	8.14	1.10	1.48	1.58	1.59	2.14	2.28
stainless steel 304 ^49^	8.00	1.18	1.58	1.68	1.71	2.27	2.43
lead^41^	11.35	0.55	0.99	1.09	0.800	1.43	1.57
tungsten^41^	19.25	0.37	0.69	0.67	0.53	0.89	0.97

When compared to Pb, a benchmark γ-ray shielding material, Hf_2_CdC demonstrates comparable or superior performance, particularly at moderate photon energies. At 1173 keV, Hf_2_CdC shows an HVL of 0.97 cm, which is slightly lower than Pb’s 0.99 cm. Similarly, at 1332 keV, the HVL of Hf_2_CdC is 1.05 cm, compared to 1.09 cm for Pb. These results indicate that Hf_2_CdC provides improved attenuation in energy ranges dominated by Compton scattering. However, at 662 keV, Hf_2_CdC shows an HVL of 0.61 cm, which is slightly higher than Pb’s 0.55 cm, reflecting Pb’s stronger attenuation performance at lower energies due to enhanced photoelectric absorption.

The observed trend where Hf_2_CdC demonstrates superior shielding at moderate energies but slightly reduced performance at lower energies compared to Pb can be explained by examining the fundamental mechanisms of γ-ray interaction with matter and their dependence on both the energy of the incident photon and the properties of the absorbing material, particularly atomic number and density. The dominant interaction mechanisms in the energy range considered include the photoelectric effect, Compton scattering, and pair production. The relative significance of each mechanism varies substantially with photon energy.

As mentioned earlier, at lower photon energies, the photoelectric effect is the dominant interaction. The probability of photoelectric absorption depends strongly on the atomic number (*Z* ^4–5^) of the absorber and is inversely proportional to the cube of the photon energy (*E* ^3^).^55^ The approximate relationship for the linear attenuation coefficient due to the photoelectric effect (μ_pe_) is given by

11 $$\mu_{\mathrm{pe}}\propto \rho \frac{Z^{4-5}}{{\mathrm{AE}}^{3}}$$

where μ_pe_ is the linear attenuation coefficient for the photoelectric effect, ρ is the material’s density, *Z* is the atomic number, *A* is the atomic mass, and *E* is the photon energy.

This strong *Z* dependence explains why materials with high atomic numbers, such as Pb (*Z* = 82), are particularly effective at attenuating low-energy γ rays via the photoelectric effect. In this energy regime, Pb outweighs the Hf_2_CdC, resulting in slightly better performance against the 662 keV photons emitted by ^137^Cs.

As the photon energy increases, Compton scattering becomes the predominant interaction mechanism. The linear attenuation coefficient for Compton scattering (μ_C_) is approximately proportional to the material’s atomic number (*Z* ^1^) and the density, with its energy dependence described by the Klein–Nishina differential cross-section

12 $$\mu_{C}\propto \rho \frac{Z}{A}\int \mathrm{KN}\left(E\right)$$

As the photon energy increases beyond 1.022 MeV, pair production becomes a contributing interaction mechanism. The linear attenuation coefficient for pair production (μ_pp_) is approximately proportional to the square of the atomic number (*Z* ^2^) and the material’s density, expressed as

13 $$\mu_{\mathrm{pp}}\propto \rho \frac{Z^{2}}{A}\int \mathrm{pp}\left(E\right)$$

At higher energies (around the ^60^Co energies of 1173 and 1332 keV), although the atomic number remains relevant, its influence becomes less dominant than in the photoelectric region. In this regime, the material’s density becomes a more critical determinant of attenuation effectiveness.

It is important to note that, while Pb has a higher atomic weight (207.2 u) than any of the elements constituting Hf_2_CdC (Hf: 178.49 u, Cd: 112.41 u, C: 12.01 u), it crystallizes in a face-centered cubic (FCC) structure with a lattice parameter of approximately 4.95 Å and a unit cell volume of about 121.5 Å^3^, containing four atoms per unit cell. The FCC structure, though relatively efficient in atomic packing, is characterized by metallic bonding, which permits larger interatomic distances and introduces some inherent spatial inefficiency.

In contrast, Hf_2_CdC adopts a near-close-packed hexagonal structure characteristic of MAX phases, with lattice parameters of *a* = 3.176 Å and *c* = 14.453 Å, resulting in a unit cell volume of approximately 127 Å^3^. Although its constituent elements have lower atomic weights than Pb, the structure contains eight atoms per unit cell, including interstitial carbon atoms occupying specific crystallographic sites. These carbon atoms form strong covalent bonds with the transition metal Hf, while metallic bonding occurs between Hf and Cd, creating a compact, mixed-bonding framework. This combination of increased atomic occupancy, strong directional bonding, and efficient space utilization leads to a higher mass per unit volume in Hf_2_CdC compared to Pb. Consequently, Hf_2_CdC exhibits a bulk density of 12.67 g·cm^–3^, which surpasses the density of Pb at 11.34 g·cm^–3^. This higher density results in a greater number of electrons per unit volume, enhancing the probability of interactions through Compton scattering and pair production. Therefore, despite having constituent elements with lower atomic numbers than Pb, the superior density of Hf_2_CdC enables it to achieve a lower HVL at higher photon energies.

Nonetheless, it is worth stressing that very high-density materials such as pure tungsten (density 19.25 g/cm^3^) exhibit even lower HVL and MFP values, as shown in Table 3. While tungsten offers superior γ-ray attenuation overall, Hf_2_CdC remains highly competitive, especially when considering its multifunctional potential for broader radiation shielding applications and the unique properties inherent to MAX phases.

In practical radiation shielding applications, the interaction of photons with matter extends beyond simple attenuation to include significant scattering events that contribute to the buildup of secondary radiation within the material. The EABF quantifies this phenomenon by representing the ratio of the total energy absorbed in a material (due to both primary and scattered photons) to the energy absorbed from the unscattered primary beam. Understanding EABF is crucial, particularly in broad-beam geometries and for thicker shielding materials where multiple scattering events are likely.

Figure 5a–c illustrate the energy dependence of the EABF for Ti_2_CdC, Zr_2_CdC and Hf_2_CdC, respectively, at various penetration depths ranging from 1 to 40 MFP. A consistent trend across all three MAX phases is a general increase in EABF with increasing penetration depth throughout the investigated energy range. This behavior results from the higher probability of multiple scattering events as photons travel deeper into the material, leading to a greater accumulation of secondary photons and, consequently, enhanced energy absorption.

**Figure 5:**
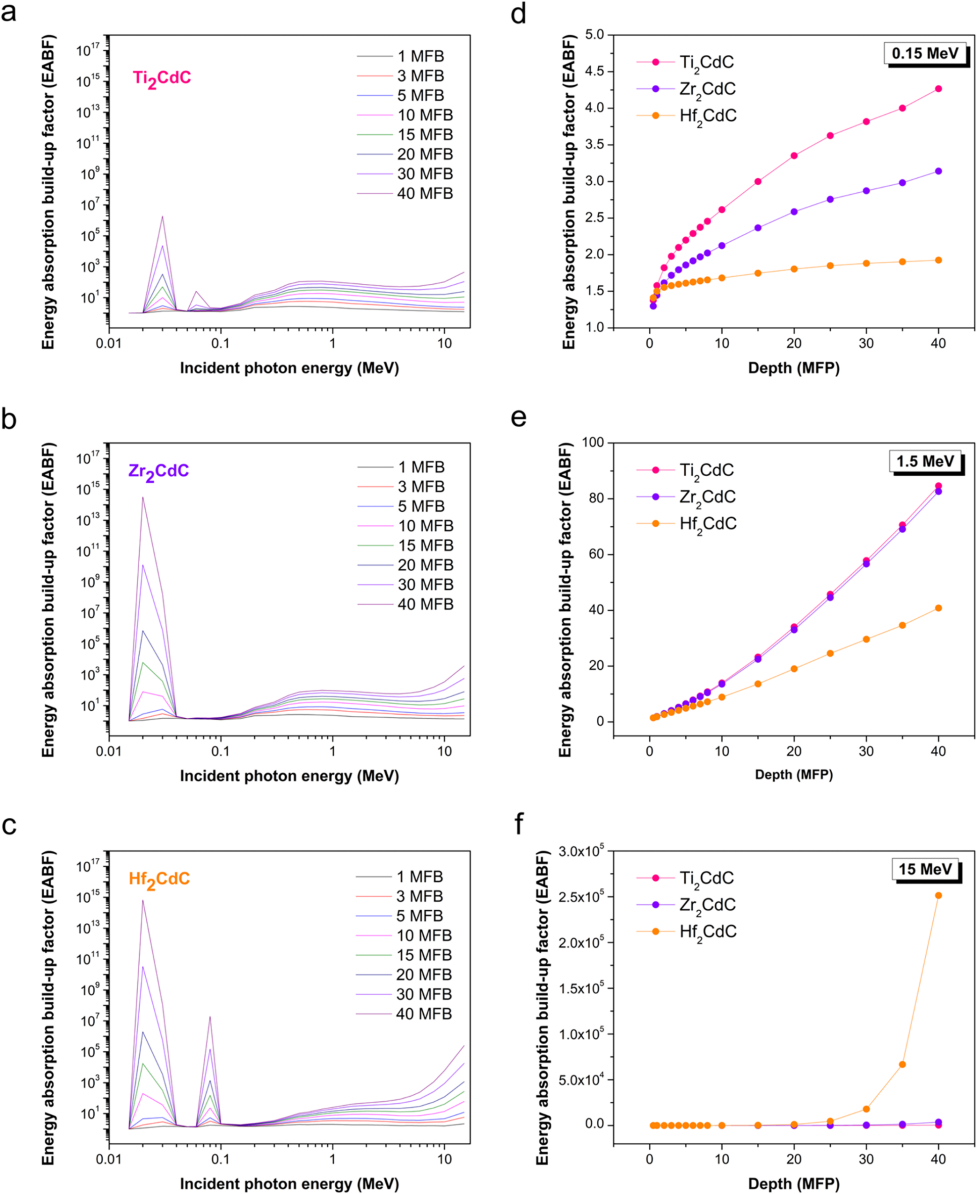
Energy absorption build-up factor for (a) Ti_2_CdC, (b) Zr_2_CdC, and (c) Hf_2_CdC at various mean free paths ranging from 1 to 40, within the 15 keV to 15 MeV energy range. The variations of energy absorption build-up factor as a function of penetration depths for (d) 0.15 MeV, (e) 1.5 MeV, and (f) 10 MeV energies.

The overall energy dependence of the EABF for the investigated MAX phases, as depicted in Figure 5a–c, exhibits trends consistent with those reported in the literature for various shielding materials containing heavy elements.^56,57^ In the low-energy region (0.01 MeV to approximately 0.1 MeV), distinct peaks appear in the EABF plots due to the K-absorption edges of the constituent elements. Aside from these peaks, EABF values in this low-energy range remain relatively low, particularly at shallower depths. This is attributed to the high probability of initial photon absorption, which limits deep penetration and multiple scattering.

In the intermediate energy region (approximately 0.1 MeV to a few MeV), where Compton scattering is the dominant interaction mechanism, the EABF demonstrates a more gradual increase with increasing photon energy and penetration depth. Scattered photons in this energy range contribute significantly to the total absorbed energy, resulting in a noticeable buildup effect.

At higher photon energies (above a few MeV), where pair production becomes increasingly significant, the EABF continues to rise, particularly at larger penetration depths. Notably, the rate of EABF increase at higher photon energies (above 6 MeV) is more pronounced for Hf_2_CdC at greater depths compared to Zr_2_CdC and Ti_2_CdC. The dependence of EABF on penetration depth at selected energies (Figure 5d–f) provides further insight into the shielding characteristics of MAX phases. At 0.15 MeV (Figure 5d), Ti_2_CdC exhibits the highest EABF, indicating a greater contribution from scattered photons relative to the primary absorbed energy when compared to Zr_2_CdC and Hf_2_CdC. At 1.5 MeV (Figure 5e), the EABF increases more linearly with depth for all three materials, with Ti_2_CdC and Zr_2_CdC exhibiting similar buildup values, both higher than that of Hf_2_CdC. Contrarily, the trend at 10 MeV (Figure 5f) reveals a significant shift. Hf_2_CdC exhibits a pronounced increase in EABF at greater penetration depths, surpassing both Zr_2_CdC and Ti_2_CdC. This observation aligns with previous findings, where heavier atoms (e.g., Mo, W, Pb, Bi) tend to exhibit increased EABF’s at higher energies, likely due to enhanced pair production and secondary interaction processes.^58^

To complement the γ-ray shielding analysis, a comparative study of charged particle (proton, α, and electron) interactions with M_2_CdC (M = Ti, Zr, Hf) was conducted. Proton interactions with these M_2_CdC compounds, as shown in Figure 6, reveal the electronic stopping power, nuclear stopping power, total stopping power, and projected range as functions of proton kinetic energy. Energetic protons lose energy primarily through electronic interactions (such as ionization and excitation of target electrons) and nuclear interactions involving elastic scattering from target nuclei.

**Figure 6:**
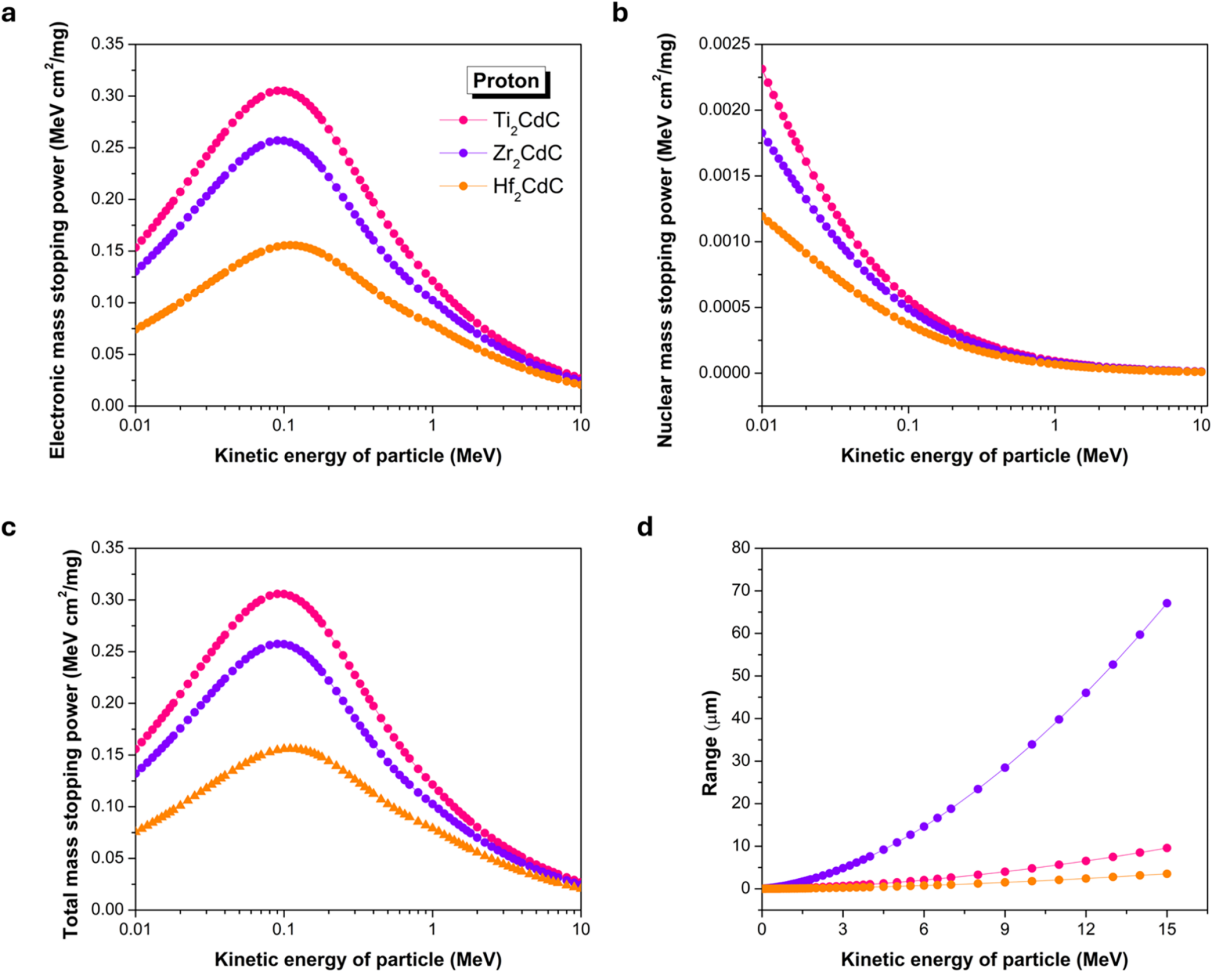
Comparison of mass stopping power and range for protons interacting with Ti_2_CdC, Zr_2_CdC, and Hf_2_CdC. (a) electronic stopping power, (b) nuclear stopping power, (c) total stopping power, and (d) range.

As shown in Figure 6a,b, the electronic mass stopping power values are significantly higher than nuclear mass stopping power values across the entire energy spectrum. This confirms that electronic interactions are the dominant mechanism by which energetic protons lose energy in these materials. For instance, at 0.1 MeV, the electronic mass stopping power for Ti_2_CdC is approximately 0.3051 MeV·cm^2^/mg, whereas its nuclear mass stopping power is around 0.00056 MeV·cm^2^/mg. Consequently, the total mass stopping power (Figure 6c) is predominantly governed by the electronic component, causing Figure 6a,6c to appear very similar in both shape and magnitude.

The plots of total mass stopping power (Figure 6c) exhibit the characteristic Bragg peak, where the stopping power initially increases with proton kinetic energy, reaching a maximum around 0.09–0.1 MeV, and then gradually decreases at higher energies. This decrease beyond the peak is consistent with the Bethe formula, where stopping power scales inversely with the square of the proton’s velocity (*v* ^2^) and energy (*E*). At the Bragg peak, the order of total mass stopping power is Ti_2_CdC (0.3058 MeV·cm^2^/mg) > Zr_2_CdC (0.2575 MeV·cm^2^/mg) > Hf_2_CdC (0.1558 MeV·cm^2^/mg).

Although Hf_2_CdC consistently exhibits the lowest total mass stopping power (*S*_tot_/ρ) across the entire energy range (as seen in Figure 6c), its projected proton ranges (Figure 6d) reveal a contrasting trend. Hf_2_CdC yields the shortest proton range: at 10 MeV, the proton ranges are 1.77 μm for Hf_2_CdC, 4.8 μm for Ti_2_CdC, and 33.92 μm for Zr_2_CdC. This trend can be explained by considering the linear stopping power (*S*), which is the product of mass stopping power and material density (*S* = (*S*/ρ) × ρ), and is inversely related to the projected range.

Hf_2_CdC, having the highest density among the three materials, achieves a much higher linear stopping power despite its lower mass stopping power. As a result, it provides the most effective proton attenuation per unit thickness. Nonetheless, the observed range order of Hf_2_CdC < Ti_2_CdC < Zr_2_CdC suggests that while Hf_2_CdC’s density plays a dominant role, the interplay between mass stopping power and density also allows Ti_2_CdC (despite its lower density compared to Zr_2_CdC) to achieve a higher linear stopping power than Zr_2_CdC.

Similar principles govern the interaction of α particles with the M_2_CdC compounds, as shown in Figure 7. α particles (He^2+^), being significantly heavier and carrying twice the charge of protons (H^+^), interact more intensely with matter. Like protons, α particles primarily lose energy via electronic interactions, with electronic mass stopping power (Figure 7a) being considerably larger than nuclear mass stopping power (Figure 7b) across most of the energy spectrum. The total mass stopping power for α particles (Figure 7c) also exhibits a Bragg peak, which occurs at a higher energy (around 0.65–0.9 MeV) than that observed for protons. The magnitude of total mass stopping power at the peak is substantially higher for α particles compared to protons. This is primarily because electronic stopping power, according to the Bethe formula, is proportional to the square of the projectile’s charge (*Z*_proj_^2^). Since α particles have *Z*_proj_ = +2*e* (*Z*_proj_^2^ = 4), they lose energy approximately four times more rapidly than protons at the same velocity, leading to significantly larger mass stopping power values. At their respective peaks, the order of total mass stopping power for α particles mirrors that of protons: Ti_2_CdC (0.8905 MeV·cm^2^/mg) > Zr_2_CdC (0.7961 MeV·cm^2^/mg) > Hf_2_CdC (0.4889 MeV·cm^2^/mg). Consequently, the projected ranges of α particles (Figure 7d) are much shorter than those of protons at comparable energies. At 10 MeV, α particle ranges are approximately 0.27 μm for Hf_2_CdC, 0.5 μm for Ti_2_CdC, and 3.7 μm for Zr_2_CdC. As with the proton results, Hf_2_CdC shows the shortest range values due to its higher density, which results in the greatest linear stopping power, followed by Ti_2_CdC and then Zr_2_CdC. The inherently short ranges of α particles in these M_2_CdC compounds indicate that even very thin layers would be highly effective in completely stopping α radiation.

**Figure 7:**
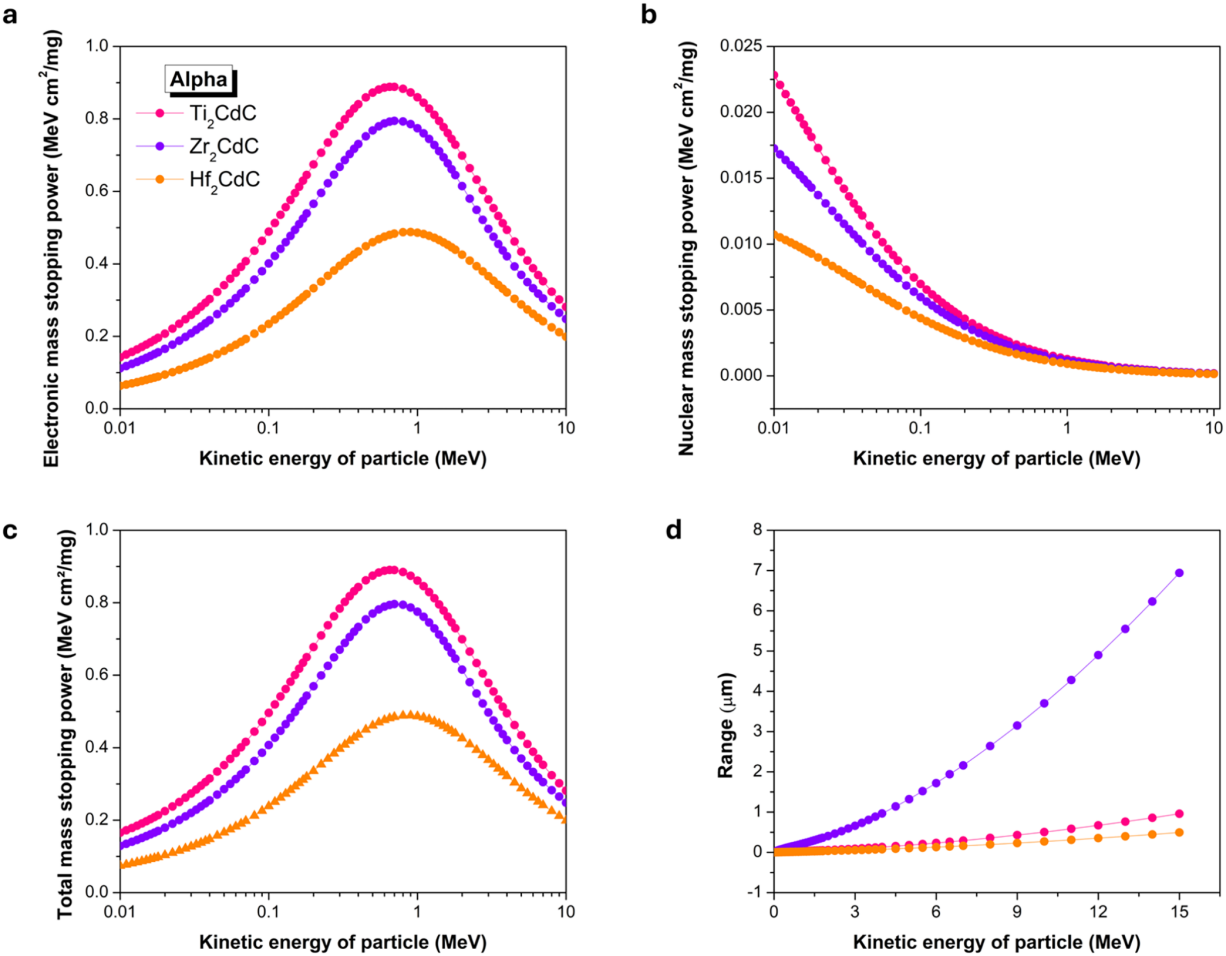
Comparison of mass stopping power and range for α particles interacting with Ti_2_CdC, Zr_2_CdC, and Hf_2_CdC. (a) Electronic mass stopping power, (b) nuclear mass stopping power, (c) total mass stopping power, and (d) range.

Shielding electrons requires accounting for two primary mechanisms of energy loss: collisional losses, arising from ionization and excitation of target atoms, and radiative losses, primarily due to bremsstrahlung (the emission of photons as electrons decelerate). Together, these mechanisms contribute to the total energy loss experienced by electrons as they traverse the material. Therefore, the total mass stopping power (*S*_tot_/ρ) for electrons is given by the sum of the mass collision stopping power (*S*_col_/ρ) and the mass radiative stopping power (*S*_rad_/ρ)

14 $$S_{\mathrm{tot}}/\rho =S_{\mathrm{col}}/\rho +S_{\mathrm{rad}}/\rho$$

Figure 8a shows that mass collision stopping power is the dominant energy loss mechanism at lower electron kinetic energies (below 1 MeV), and its magnitude generally decreases as the electron energy increases. Across the entire energy range, the mass collision stopping power consistently follows the order: Ti_2_CdC > Zr_2_CdC > Hf_2_CdC. For example, at 0.1 MeV, the mass collision stopping power is approximately 2.945 MeV·cm^2^/g for Ti_2_CdC, 2.762 MeV·cm^2^/g for Zr_2_CdC, and 2.539 MeV·cm^2^/g for Hf_2_CdC. This trend in mass collision stopping power can be explained by the fundamental expression for mass collision stopping power, derived from Bethe’s theory^59^

15 $$\frac{1}{\rho }S_{\mathrm{col}}=\frac{N_{a}Z_{\mathrm{eff}}}{A_{\mathrm{eff}}}\int W\frac{d_{\sigma }}{d_{w}}dW$$

**Figure 8:**
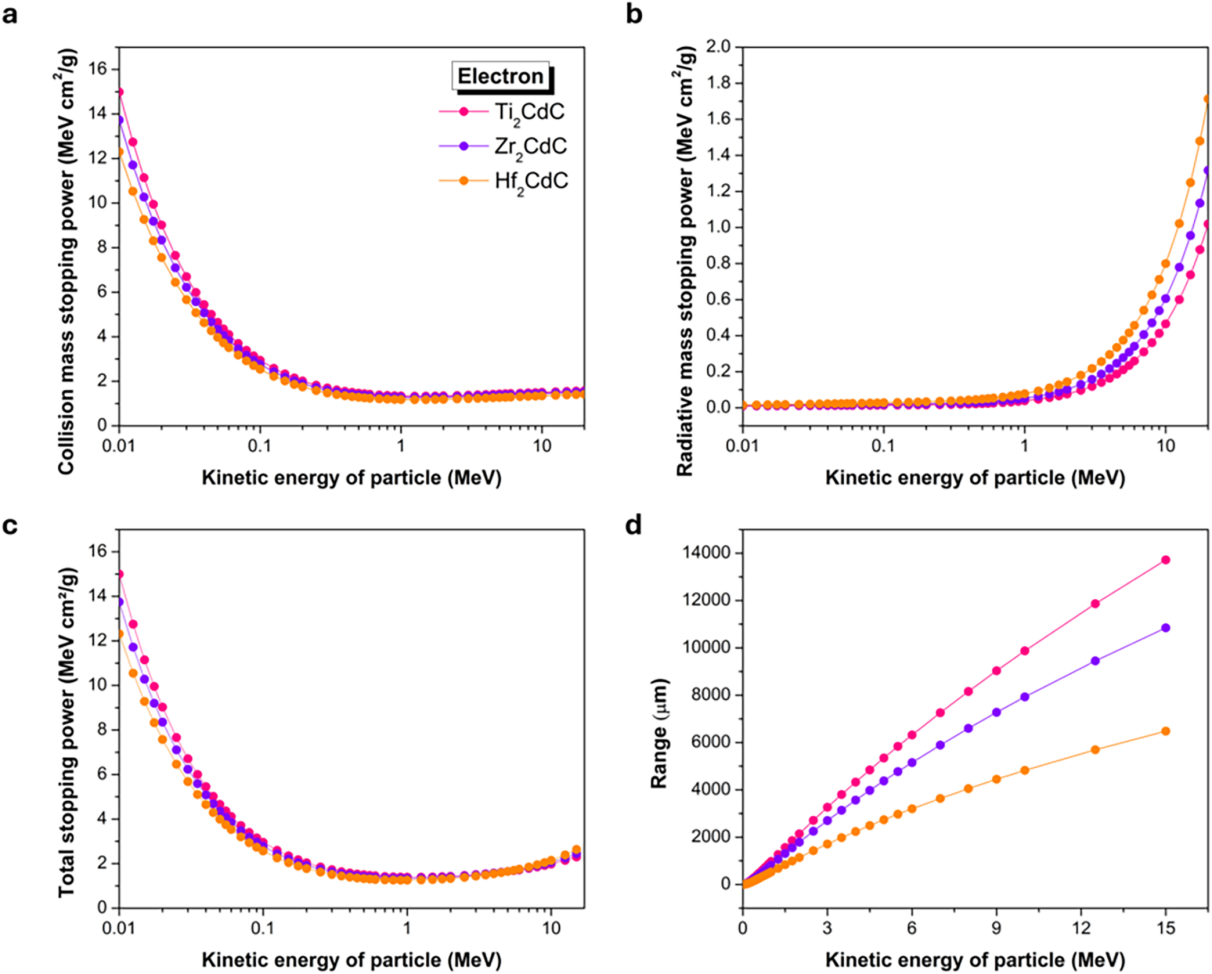
Comparison of mass stopping power and range for electrons interacting with Ti_2_CdC, Zr_2_CdC, and Hf_2_CdC. (a) Collision mass stopping power, (b) radiative mass stopping power, (c) total mass stopping power, and (d) range.

The term $\frac{N_{a}Z_{\mathrm{eff}}}{A_{\mathrm{eff}}}$ represents the number of atomic electrons per unit mass of the material, where *N*_a_ is Avogadro’s number, *Z*_eff_ is the effective atomic number, and *A*_eff_ is the effective molar mass of the compound. The integral $\int W\frac{d_{\sigma }}{d_{w}}dW$ represents the average energy transferred (*W*) in collisions, weighted by the differential cross-section $\left(\frac{d_{\sigma }}{d_{w}}\right)$ for these energy transfers.

The primary factor influencing the observed order (Ti_2_CdC > Zr_2_CdC > Hf_2_CdC) is the $\frac{N_{a}Z_{\mathrm{eff}}}{A_{\mathrm{eff}}}$ term, which is directly proportional to the effective *Z*_eff_/*A*_eff_ ratio of the material. Elements with lower atomic masses generally have a slightly higher *Z*/*A* ratio. For instance, the ratio of atomic number (*Z*) to molar mass (*A*) for the M-site elements is approximately 0.4596 mol/g for Ti (*Z* = 22, *A* ≈ 47.87 g/mol), 0.4384 mol/g for Zr (*Z* = 40, *A* ≈ 91.22 g/mol), and 0.4034 mol/g for Hf (*Z* = 72, *A* ≈ 178.49 g/mol). Consequently, Ti_2_CdC, which contains the lightest M-site element, is expected to have a more favorable overall effective *Z*_eff_/*A*_eff_ ratio. This implies that Ti_2_CdC offers more target electrons per unit mass for interactions with incoming particles compared to Hf_2_CdC.

Furthermore, the integral term, which depends on the differential cross-section for energy loss, is influenced by the mean excitation potential (*I*) of the material. Materials with higher *Z*_eff_, such as Hf_2_CdC, also have significantly larger *I* values. A larger *I* indicates that atomic electrons are, on average, more tightly bound, which can reduce the probability or efficiency of certain energy transfers, thereby tending to decrease the stopping power. Thus, while Hf_2_CdC has a higher overall *Z*_eff_ per atom, the combination of a less favorable *Z*_eff_/*A*_eff_ ratio (contributing to fewer electrons per unit mass) and a larger mean excitation potential *I* leads to its lower mass collision stopping power for electrons across the energy range studied when compared to Ti_2_CdC and Zr_2_CdC.

Conversely, radiative stopping power, shown in Figure 8b, arises from bremsstrahlung (the emission of photons as electrons decelerate in the electric field of atomic nuclei). Radiative stopping power increases with electron energy and is strongly dependent on the target material, being roughly proportional to the square of the effective atomic number (*Z*_eff_^2^) and the incident electron energy.^60^

Consequently, Hf_2_CdC, with the highest *Z*_eff_, exhibits the highest radiative stopping power, followed by Zr_2_CdC and Ti_2_CdC, particularly above 1 MeV. For example, at 10 MeV, radiative stopping power values are 0.7998 MeV·cm^2^/g for Hf_2_CdC, 0.6064 MeV·cm^2^/g for Zr_2_CdC, and 0.4657 MeV·cm^2^/g for Ti_2_CdC.

The total mass stopping power for electrons (Figure 8c), being the sum of collision and radiative stopping power, reflects these opposing trends. At low energies, total mass stopping power is higher for Ti_2_CdC due to collision stopping power dominance. As energy increases, total mass stopping power values for all materials decrease, reaching a minimum around 1 MeV. Beyond this, total mass stopping power rises due to the increasing contribution of radiative stopping power. This behavior means that the total mass stopping power curve for electrons typically shows a decrease at lower energies, potentially reaching a minimum before rising at higher energies where radiative losses dominate. In the higher energy region (above 5 MeV), Hf_2_CdC exhibits the highest total mass stopping power due to the *Z*_eff_^2^ dependence of bremsstrahlung. For instance, at 15 MeV, approximate total mass stopping power values are 2.637 MeV·cm^2^/g for Hf_2_CdC, 2.445 MeV·cm^2^/g for Zr_2_CdC, and 2.283 MeV·cm^2^/g.

The projected ranges for electrons, calculated as the continuous slowing down approximation range, are depicted in Figure 8d. It is important that the continuous slowing down approximation range represents an average path length; unlike heavy particles, electrons undergo significant angular scattering (multiple scattering) due to their low mass, resulting in tortuous paths. Therefore, the continuous slowing down approximation range may not directly correspond to the straight-line penetration depth, which is often shorter. Consistent with findings for heavy particles where high density contributes to shorter ranges, Hf_2_CdC offers the shortest electron range, followed by Zr_2_CdC, and then Ti_2_CdC. For example, at 15 MeV, the electron ranges are approximately 6481 μm for Hf_2_CdC, 10846 μm for Zr_2_CdC, and 13717 μm for Ti_2_CdC. The superior shielding performance of Hf_2_CdC against electrons, especially at higher energies, is due to its higher density (which enhances the effect of stopping power per unit mass) and its significantly greater radiative stopping power.

Neutrons, being uncharged particles, interact with matter through nuclear rather than electromagnetic forces, making their behavior fundamentally different from photons or charged particles. These interactions depend heavily on the target material and can be complex. While neutron emission commonly originates from nuclear reactors via fission of uranium or plutonium, various other sources such as spontaneous fission (e.g., from ^252^Cf or ^244^Cm), (α, *n*) sources (e.g., PuBe, AmBe), (γ, *n*) reactions, light ion accelerators (e.g., D-T, D-D fusion), and spallation sources are also encountered in scientific, industrial, and medical contexts. Effective neutron shielding typically requires a two-stage process: first, moderation of fast neutrons by light nuclei to reduce their energy, followed by absorption of thermalized neutrons using materials with high neutron capture cross sections. Additionally, neutron capture often leads to secondary γ emission, necessitating further γ attenuation.^61–63^

The neutron shielding capabilities of the M_2_CdC (M = Ti, Zr, Hf) compounds were assessed by examining their FNRCS. The FNRCS is a macroscopic parameter that reflects the probability per unit path length of a fast neutron undergoing an initial interaction within the material such as absorption, or elastic/inelastic scattering that effectively removes it from the incident, uncollided beam. Superior shielding against fast neutrons per unit thickness is indicated by a higher FNRCS value. The following relation was used to calculate the FNRCS using the mixture rule

16 $$\sum_{R}={\sum_{i}\rho_{i}{(\Sigma }_{R}/\rho )}_{i}$$

where ρ_*i*_ is the partial density of the *i*th constituent, and (∑_*R*_/ρ)_i_ is the mass removal cross-section.

Among the studied compounds, Hf_2_CdC exhibited the highest fast neutron removal cross-section (FNRCS) of 0.163 cm^–1^, outperforming Zr_2_CdC (0.125 cm^–1^) and Ti_2_CdC (0.115 cm^–1^). This indicates that Hf_2_CdC is the most effective among the three MAX phase compounds for fast neutron shielding. Interestingly, this result contrasts with the trend observed in the individual neutron mass removal cross sections of the M-site elements (Ti, Zr, and Hf), which decrease with increasing atomic number: Ti (0.02152 cm^2^/g) > Zr (0.01431 cm^2^/g) > Hf (0.01035 cm^2^/g). This behavior aligns with the principle that neutrons, being light particles, transfer more energy during collisions with nuclei of similar mass. Despite Hf atoms having the lowest individual neutron removal cross-section among the three, Hf_2_CdC superior FNRCS is primarily attributed to its higher density, as described by the governing relationship in eq 6, where FNRCS scales with material density.

Moreover, when compared to common neutron shielding materials, Hf_2_CdC’s FNRCS (0.163 cm^–1^) significantly surpasses that of graphite (0.077 cm^–1^), paraffin (0.0773 cm^–1^), concrete (0.094 cm^–1^), B_4_C (0.141 cm^–1^), water (0.1023 cm^–1^), and borate glasses (0.111 cm^–1^) and is notably higher than Pb (0.118 cm^–1^).^17,64,65^

Beyond fast neutron interactions, the ability of a material to absorb thermal neutrons is equally crucial for comprehensive neutron shielding. Notably, all M_2_CdC compounds exhibit exceptional thermal neutron absorption capability primarily due to presence of Cd in their composition. Cd, particularly its isotope ^113^Cd, possesses one of the highest thermal neutron capture cross sections among all elements, making it an extremely efficient absorber of slow neutrons. The calculated LAC values for thermal neutrons (25,4 meV) are as follows: Hf_2_CdC: 43.38 cm^–1^, Ti_2_CdC: 42.61 cm^–1^, and Zr_2_CdC: 37.79 cm^–1^. Among the three, Hf_2_CdC again demonstrates the highest attenuation capability, confirming its superior performance not only for fast neutrons but also for thermal neutron shielding.

Beyond its excellent performance in neutron moderation and absorption, Hf_2_CdC also addresses a key limitation of conventional shielding materials: the need to attenuate secondary γ radiation generated during neutron capture, particularly by Cd. Traditional shielding systems typically require an additional high-Z layer (e.g., Pb, W, Bi) to attenuate these γ rays. However, in Hf_2_CdC, the presence of Hf, with its high atomic number, is likely to fulfill this role intrinsically. It can be postulated that Hf not only contribute to the material’s high density, enhancing fast neutron attenuation, but also might play a significant role in γ-ray absorption, including both primary and secondary radiation. This multifunctional capability, combining fast neutron removal, thermal neutron capture, and inherent γ attenuation positions Hf_2_CdC as a promising “all-in-one” shielding material. Such integrated performance could eliminate the need for complex, multilayered shielding architectures, offering advantages in terms of reduced weight, volume, and fabrication complexity. These benefits are particularly relevant for space-constrained or weight-sensitive applications, such as spent nuclear fuel transport, compact reactors, medical imaging equipment, and aerospace systems.

On the other hand, across the studied energy range, Zr_2_CdC consistently shows intermediate HVL and MFP values between Ti_2_CdC and Hf_2_CdC Ti2CdC, reflecting the moderate atomic number of Zr (*Z* = 40) and the overall density of the Zr_2_CdC compound (7.63 g/cm^3^). This indicates that Zr_2_CdC provides a balanced combination of shielding efficiency and material weight, which could be advantageous in applications where extreme attenuation is not necessary.

It is worth stressing that, among the studied M_2_CdC phases, only Ti_2_CdC has been successfully synthesized to date. Jeitschko et al. prepared Ti_2_CdC by reacting stoichiometric mixtures of TiC, Ti, and Cd powders sealed in evacuated quartz ampules, followed by prolonged annealing at 750 °C. The successful synthesis of this material demonstrates the practical feasibility of producing Cd-containing MAX phases, though the volatility and toxicity of Cd require careful handling during processing. While Hf_2_CdC and Zr_2_CdC are currently only theoretically predicted to be formable, ongoing advances in powder metallurgy, molten salt, and thin-film deposition techniques may facilitate their experimental realization. Further studies on the long-term stability and mechanical integrity of these MAX-phase carbides under prolonged radiation exposure will be essential to fully realize their potential as next-generation radiation shielding materials.

## 4 Conclusions

This study comprehensively assessed the radiation attenuation capabilities of M_2_CdC (M = Ti, Zr, Hf) MAX phase carbides, revealing the exceptional potential of Hf_2_CdC as a promising multifunctional shielding material. The findings strongly demonstrate its superior performance across diverse radiation types:

- For γ-rays, Hf_2_CdC outperformed conventional Pb in terms of HVL and MFP at moderate energies of 1173 and 1332 keV where Compton scattering is the dominant interaction process, driven by its high density.
- For charged particles, Hf_2_CdC’s high density led to superior stopping power and remarkably short penetration ranges for protons and α particles, alongside effective attenuation of high-energy electrons via enhanced radiative stopping.
- In terms of fast neutron attenuation, Hf_2_CdC’s FNRCS (0.163 cm^–1^) proved highly efficient, consistently surpassing its MAX phase counterparts and a broad range of conventional shielders, including B_4_C (0.141 cm^–1^).
- Excellent thermal neutron absorption was confirmed for all M_2_CdC (M = Ti, Zr, Hf) MAX phase carbides due to Cd, with Hf_2_CdC leading with an LAC of 43.38 cm^–1^.

This broad-spectrum effectiveness is primarily attributed to Hf_2_CdC’s unique hexagonal layered crystal structure, which facilitates its high density (12.67 g/cm^3^), alongside its strategic elemental composition combining high-Z Hf (*Z* = 72), potent neutron-capturing Cd, and lighter carbon for effective fast neutron moderation. Coupled with the inherent robust engineering properties of MAX phases including high mechanical strength, thermal stability, and radiation tolerance. Hf_2_CdC emerges as a promising ’all-in-one’ solution for demanding radiological environments. Overall, this research provides insights into the comprehensive shielding capabilities of these specific MAX phases and highlights the vast potential for further exploration of the broader MAX phase family in advanced radiation protection applications.
